# Changes in Nuclear Shape and Gene Expression in Response to Simulated Microgravity Are LINC Complex-Dependent

**DOI:** 10.3390/ijms21186762

**Published:** 2020-09-15

**Authors:** Srujana Neelam, Brian Richardson, Richard Barker, Ceasar Udave, Simon Gilroy, Mark J. Cameron, Howard G. Levine, Ye Zhang

**Affiliations:** 1 Utilization and Life Sciences, NASA John F. Kennedy Space Center, Kennedy Space Center, Merritt Island, FL 32899, USA; Howard.G.Levine@nasa.gov; 2 Department of Botany, University of Wisconsin-Madison, Madison, WI 53706, USA; dr.richard.barker@gmail.com (R.B.); sgilroy@wisc.edu (S.G.); 3 Universities Space Research Association, Columbia, MD 21046, USA; 4 Department of Population and Quantitative Health Sciences, Case Western Reserve University, Cleveland, OH 44106, USA; bxr183@case.edu (B.R.); mjc230@case.edu (M.J.C.); 5 Department of Biomedical Engineering, Arizona State University, Tempe, AZ 85281, USA; ceasar.udave@gmail.com

**Keywords:** LINC complex, simulated microgravity, nuclear morphology

## Abstract

Microgravity is known to affect the organization of the cytoskeleton, cell and nuclear morphology and to elicit differential expression of genes associated with the cytoskeleton, focal adhesions and the extracellular matrix. Although the nucleus is mechanically connected to the cytoskeleton through the Linker of Nucleoskeleton and Cytoskeleton (LINC) complex, the role of this group of proteins in these responses to microgravity has yet to be defined. In our study, we used a simulated microgravity device, a 3-D clinostat (Gravite), to investigate whether the LINC complex mediates cellular responses to the simulated microgravity environment. We show that nuclear shape and differential gene expression are both responsive to simulated microgravity in a LINC-dependent manner and that this response changes with the duration of exposure to simulated microgravity. These LINC-dependent genes likely represent elements normally regulated by the mechanical forces imposed by gravity on Earth.

## 1. Introduction

The nucleus is the largest organelle of the cell and homeostatic control of its shape and position are known to be crucial for cellular functions such as cell migration, cell division and the regulation of gene expression [1,2,3,4,5]. Forces generated by the cytoskeleton and external mechanical stimuli are directly transmitted to the nuclear surface through the Linker of Nucleoskeleton and Cytoskeleton (LINC) complex [3,6,7,8,9,10,11]—a series of proteins embedded in the nuclear envelope (NE). Thus, LINC complex Nesprins on the outer nuclear membrane are connected to different cytoplasmic cytoskeletal elements and to several other force-generating motor proteins. These Nesprin family proteins also have a KASH (Klarsicht, ANC-1, Syne Homology) domain that is located in the perinuclear space and connects to the SUN (Sad1 and UNC-84) proteins of the inner nuclear membrane [12]. The SUN proteins are in turn connected to chromatin through lamins. This mechanical connection from nuclear surface to chromatin is crucial in enabling the forces from the cytoskeleton and extracellular matrix (ECM) to be transmitted to both the surface and interior of the nucleus. These forces can, therefore, alter not just nuclear morphology but also internal elements such as chromatin architecture and so lead to the modulation of mechanosensitive gene expression.

Exposure of cells to microgravity is known to lead to the reorganization of the cytoskeleton and the ECM. In addition, microgravity leads to morphological changes in both the cell and its nucleus, and to altered gene expression [13,14,15,16,17,18,19,20]—all features related to LINC complex function. The LINC complex is also known to be a critical factor in the normal formation and development of tissues, such as the breast cell acinus [21]. Simulated microgravity (SMG) achieved by clinorotation disrupts features such as cell proliferation, cell adhesion, cell cycle progression and cytokine production [13,22]. These are all LINC-regulated processes, and failure in their normal functioning has been linked to aberrant developmental regulation, such as cancer. Indeed, in addition to altering all these LINC-dependent features, SMG also suppresses tumor cell growth and development in e.g., breast epithelial cell cultures [23]. However, despite such circumstantial evidence, the role of the LINC complex in cellular responses to altered gravitational environments has yet to be studied. Therefore, we have asked how the LINC complex affects changes in nuclear shape and differential gene expression in SMG. We report that the nucleus requires an intact LINC complex for its shape to be modulated by altered gravity conditions. We also identify LINC-dependent genes associated with this response, indicating that elements of the genome regulation normally seen in altered gravity requires an intact LINC complex.

## 2. Results

### 2.1. Simulated Microgravity Elicits Changes in Nuclear Shape that Depend on the LINC Complex 

As noted above, breast epithelial cells have been previously shown to exhibit responses to SMG, suggesting that they might be a useful model to study the involvement of the LINC complex in SMG-related effects. Furthermore, we have a deep understanding of nuclear shaping under normal gravity conditions in MCF 10A—a non-tumerogenic human mammary epithelial cell line [24]. This cell line was engineered to express a construct that interferes with LINC complex function (see below), making it well-suited to investigating the role of the LINC complex in SMG-related effects. Therefore, we chose MCF 10A cells, wildtype and engineered, to study how SMG may impact nuclear events, using a 3-D clinostat to simulate microgravity. 

We first established whether the nuclear shape of MCF 10A cells is responsive to clinostat-induced SMG. After SMG treatment, we fixed the cells, and the nuclei were stained with Hoechst 33342, allowing us to measure any change in nuclear height, major axis and minor axis using confocal microscopy. Actin staining was used to define the shape of the cells to ensure only those with the morphology characteristic of adherence to the substrate were analyzed. We observed that the nuclear height significantly (*p* < 0.05, *t*-test) decreased after 20 h of exposure to SMG, and the nuclear spreading area increased (Supplementary Figure S1A,B).

To understand if this change in nuclear shape in response to SMG depends on the LINC complex, we used a doxycycline inducible MCF 10A cell line where the LINC complex function is disrupted. This inducible cell line stably expresses SS-HA-SUN1L-KDEL (signal sequence-hemaglutinin epitope tagged to SUN1 protein, with a lumenal domain-ER retrieval amino acid sequence; SUN1L-KDEL) [25]. SUN1L-KDEL acts in a dominant negative fashion disrupting the LINC complex by interfering with the connection between the endogenous KASH domain of nesprin-2 and nesprin-4 proteins and the SUN1 proteins in the nuclear envelope [3,21,25]. Cells expressing a similar doxycycline inducible construct SS-GFP-KDEL (GFP-KDEL) were used as the corresponding paired control. We confirmed that the induction of SUN1L-KDEL but not GFP-KDEL did indeed disrupt the LINC complex by immunostaining with a Nesprin-2 antibody (Supplementary Figure S2). As noted above, Nesprin-2 localizes to the nuclear membrane in a LINC-complex-dependent fashion, and the immunofluorescence staining showed a loss of Nesprin-2 in the NE in the doxycycline induced SUN1L-KDEL cells but not in the corresponding control doxycycline-treated GFP-KDEL cells. These observations are consistent with those of Zhang et.al. who previously showed that this system is effective in disrupting the LINC function [21]. 

The doxycycline-induced cells were, therefore, exposed to 3-D clinorotation for 20 h. The 1G (ground) controls (i.e., identically doxycycline treated cells but not on the clinostat) were placed next to the clinostat in the same incubator. After treatment, the cells were fixed and the 3-dimensional nuclear morphology was monitored using confocal microscopy as described earlier. 

**Figure 1:**
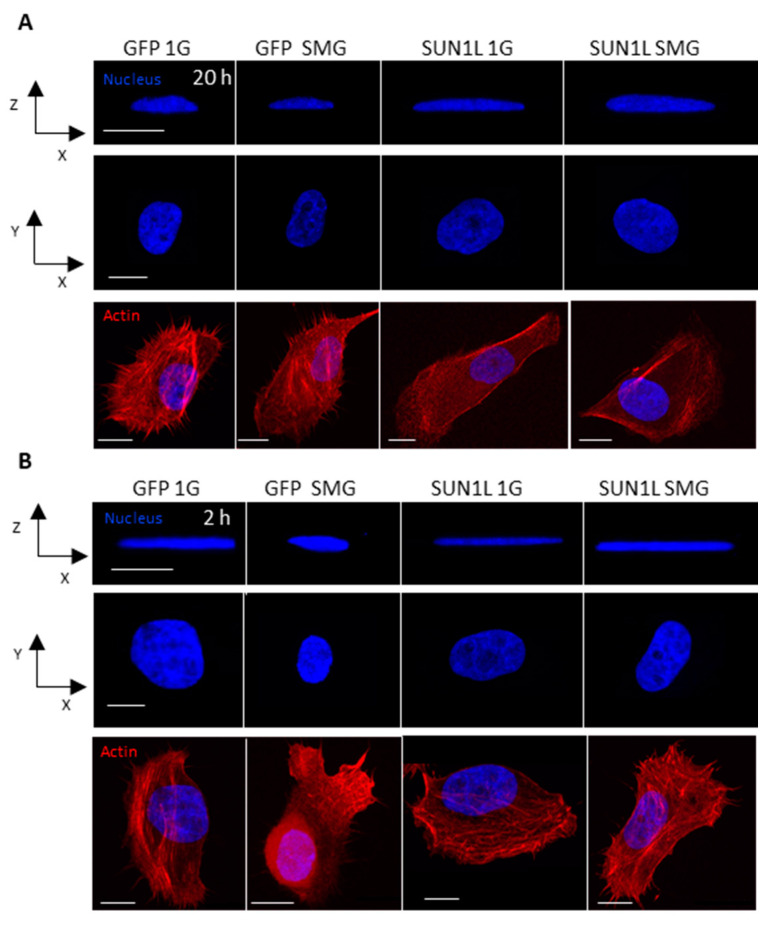
Nuclear shape response to simulated microgravity (SMG) depends on the LINC complex. (**A**) Confocal images show the nuclei (blue) and actin (red) staining (to reveal the cell morphology) of fixed GFP-KDEL (GFP) and SUN1L-KDEL (SUN1L) cells after 20 h in 1G and SMG. Top row shows the x-z view and the bottom rows show the x-y view of the corresponding nuclei and cells. Similarly, (**B**) shows confocal images of the nuclei and actin of fixed GFP-KDEL and SUN1L-KDEL cells in 1G and SMG condition for 2 h. Scale bars are 10 µm. Images are representative of the mean values.

The GFP-KDEL cells showed a decrease in nuclear height after 20 h (similar to the MCF 10A wild type cells, Supplementary Figure S1A,C,D) in SMG (Figure 1A and Table 1). In contrast, the SUN1L-KDEL cells showed a significant nuclear height increase when compared to 1G cells over this same time period in SMG. These results show that the normal nuclear shape change to SMG requires an intact LINC complex.

We next asked whether significant LINC-dependency of nuclear responses to SMG could also be seen with a shorter SMG treatment. Therefore, to observe the impact of short duration exposure of LINC disrupted cells to SMG, we assayed responses after 2 h on the clinostat. The nuclear height increased significantly (*p* < 0.05, *t*-test), and the nuclear area decreased in GFP-KDEL cells exposed to SMG for 2h. This impact, however, was opposite to the response at the 20 h time point, i.e., decrease in nuclear height in SMG (Figure 1B, Table 1), indicating that the cells may start adapting to SMG differently at longer timepoints. The SUN1L-KDEL cells on the other hand did not show any significant difference in nuclear morphology between the 1G and SMG cells over 2 h of SMG (Table 1). These observations again suggest that the effects of SMG on nuclear shape required an intact LINC complex. 

### 2.2. LINC-Dependent Differential Gene Expression in Simulated Microgravity

**Figure 2:**
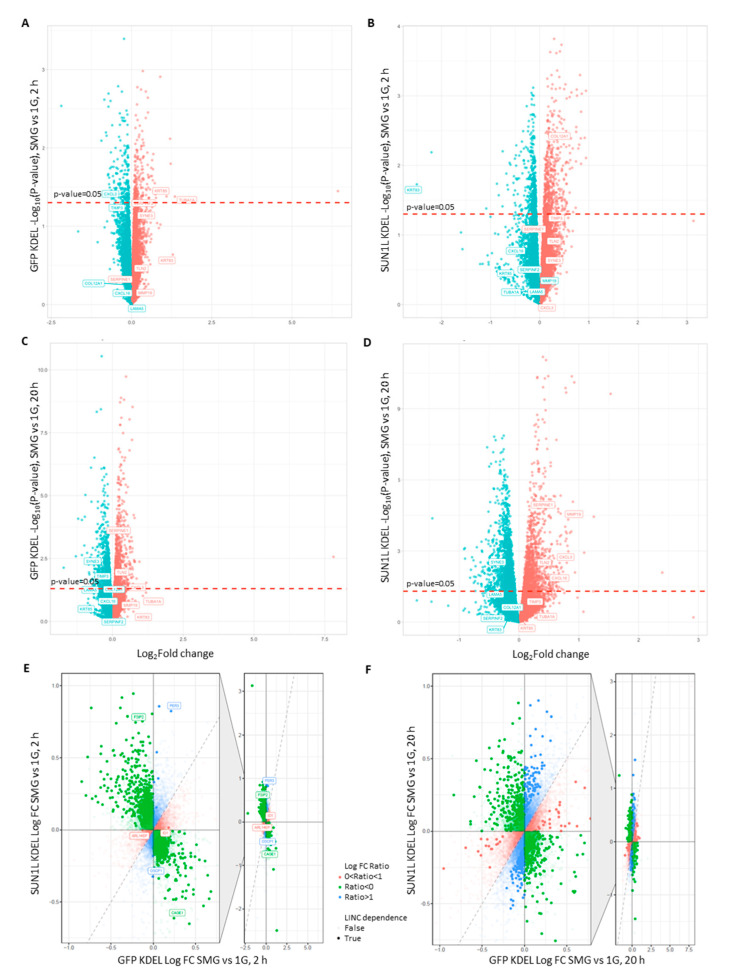
Transcriptome responds to SMG and varies upon LINC disruption at different exposure times. Volcano plots of differentially expressing genes between SMG and 1G with statistical significance in the *y*-axis and fold change on the *x*-axis in (**A**) GFP-KDEL cells after 2 h, (**B**) SUN1L-KDEL cells after 2 h, (**C**) GFP-KDEL cells after 20 h, (**D**) SUN1L-KDEL cells after 20 h. Upregulated genes are shown in orange and downregulated genes are shown in blue. Significantly differentially expressing genes with *p* ≤ 0.05 are above the red dashed line. (**E**,**F**) summarize all the LINC-dependent genes identified through the interaction model at 2 and 20 h, respectively, which are described in Supplementary Tables S3 and S6. The whole distribution is shown to the right with the indicated area magnified on the left. Genes with statistical significance (*p* < 0.05) are shown to be differentially regulated under the interaction of LINC disruption and SMG conditions and are highlighted with LINC dependence equal to True in the figure. Genes that are not highlighted (False) did not attain significance and are not dependent on LINC. For each gene, the log fold-change (logFC) for response to SMG vs. its 1G control in the SUNL-KDEL cells is divided by the same characterization but in the GFP KDEL controls and is color-coded on the plot. Based on this logFC ratio, these genes could be characterized as being regulated in the opposite direction for each cell line (green, Ratio < 0), regulated more strongly under the SUN1L KDEL cell line (blue, Ratio > 1) or regulated more strongly under the GFP cell line (pink, 0 < Ratio < 1).

Since the changes in nuclear shape triggered by both 2 and 20 h SMG appear to require the LINC complex, we hypothesized that disruption of the LINC complex with SUN1L KDEL expression might also affect patterns of gene expression. We, therefore, used RNAseq analysis to characterize differential gene expression in GFP-KDEL and SUN1L-KDEL in SMG vs. 1G controls. Figure 2A shows a plot of the fold-change in expression level (SMG vs. 1G control) against significance of this change (*p*-value) in our initial analysis of transcriptional responses after 2 h of exposure to SMG. A larger number of genes were up- or down-regulated in SUN1L-KDEL cells in SMG (558 up, 434 down, nominal *p* < 0.05) compared to those in the GFP-KDEL cells (203 up, 179 down, nominal *p* < 0.05; Figure 2A,B, Supplementary Tables S1 and S2). 

We identified the top 50 most significantly differentially expressed genes (selected as showing the smallest p-value when comparing 2 h SMG to 1G control) in each condition, which are presented in Supplementary Figure S3. Nassef et al. used true microgravity (10^-2^ G) in parabolic flight to show that the expression of genes related to the cytoskeleton, extracellular matrix and chemokines is affected in breast epithelial cells exposed to true microgravity [14]. Thus, this study provides some specific genes as well as broad classes of potential genes of interest. We, therefore, closely compared the response of the individual genes in the Nassef et al. study with their responses in our study (Supplementary Table S10). Even though we did not see similar response in any of these genes after 2 h exposure to SMG, we found that TIMP1, SERPINE1 and RDX genes were differentially regulated after 20 h SMG exposure, similar to those reported in the Nassef study in cells after experiencing 31 parabolas. The differences in gene expression may be due to the alternating hypergravity and microgravity phases experienced during each parabola. However, in our 2 h SMG data, we did see changes in genes reflecting the broad classes of gene families reported by Nassef et al. Thus, in GFP-KDEL expressing cells, 2 h SMG led to the modulation of genes associated with the cytoskeleton (e.g., KRT85, TUBA1A), the extracellular matrix (TIMP3, SERPINF2), and chemokine response (CXCL3). These genes are highlighted in Figure 2A,B. To infer whether the LINC complex might play a role in regulating the expression of any of these genes in SMG, we compared their levels in the GFP-KDEL controls to those where the LINC complex was disrupted by SUN1L-KDEL expression. We predicted that genes for which the LINC function played a role in regulating their expression under SMG conditions would show one of several patterns of response. Thus, SMG-responsive genes for which the change in transcript level is LINC-dependent might show alteration in expression in SMG in the GFP KDEL control cells but lose this responsiveness to SMG in the SUN1L KDEL lines. Some genes might show responsiveness to SMG in the control cells but show a significantly larger or smaller change in the LINC-disrupted case, implying a role of the LINC complex in the degree of repression or activation of expression. Additionally, some genes may show no differential expression in SMG when comparing GFP KDEL cells in SMG to their 1G controls, but these genes would show differential expression in the LINC-disrupted SUN1L KDEL expressing cells. In this latter case, normal gene expression homeostasis would require LINC action, and in the LINC-disrupted cells, this regulation would be lost. Loss of this control would lead to either up- or down-regulation of their transcription, specifically in SMG, revealing a further layer of LINC-dependency to their normal transcriptional regulation under SMG conditions. Indeed, our analysis shows that the differential expression of some of these genes in SMG appears to depend on an intact LINC complex. For example, the cytoskeletal element keratin (KRT85) was upregulated by 2.13-fold in SMG in GFP-KDEL cells, but there was no significant difference in its expression levels in LINC-disrupted SUN1L-KDEL cells exposed to equivalent SMG treatment. Similarly, the chemokine response marker CXCL3 (C-X-C Motif Chemokine Ligand 3) was downregulated 1.4-fold in GFP-KDEL, but this gene lost its sensitivity to SMG in SUN1L-KDEL cells. In contrast, the LINC disruption using SUN1L-KDEL resulted in the decreased expression (5.65-fold) of KRT83 (another cytoskeletal gene belonging to the keratin family), whereas this gene did not alter due to SMG in GFP-KDEL cells. Furthermore, the ECM gene (COL12A1) showed no significant difference in GFP-KDEL cells but was upregulated by 1.22-fold in the SUN1L-KDEL expressing cells. Thus, the LINC complex appears to play roles in normally inducing some genes, while repressing other genes in response to SMG and in maintaining homeostatic control of other genes under SMG conditions.

To elucidate the role of the LINC in differential gene expression, we used an interaction model between transcriptional changes seen in SUN1L-KDEL and GFP-KDEL expressing cells with and without SMG exposure. The interaction effect is described in Figure 2E where the relative fold changes between SMG and 1G in GFP-KDEL and SUN1L-KDEL samples at 2 h are compared statistically. The majority of the LINC-dependent genes reversed their direction of change upon LINC disruption (Ratio < 0). For example, the FSIP2 gene was significantly down-regulated in GFP-KDEL cells in SMG. However, the change in expression level of this gene reversed in SUN1L-KDEL cells, with a significant upregulation. Similarly, CAGE1, a gene widely associated with cancer [26] was significantly upregulated in the GFP-KDEL cells, while it was significantly downregulated in SUN1L-KDEL cells illustrating an opposite pattern of response of the gene FSIP2 to LINC disruption. In addition to these genes, we identified genes whose sensitivity to SMG was lost (0 < Ratio < 1) and genes which gained such sensitivity (Ratio > 1) upon LINC disruption. For example, ARL14EP and ID1 are the genes which were significantly differentially expressed in GFP-KDEL cells exposed to 2h of SMG but were not altered in SUN1L-KDEL cells exposed to SMG, implying that these genes lost their sensitivity to SMG upon LINC disruption. On the other hand, PER3 and OSCP1 genes were not affected by SMG in GFP-KDEL cells but were significantly differentially expressed in SUN1L-KDEL cells exposed to 2 h of SMG. The results both imply that the LINC complex is required to maintain transcriptional homeostasis of some genes in the presence of SMG and that an intact mechanical nucleo-cytoskeletal linkage may mediate response to SMG and to regulate subsequent differential gene expression of other genes.

**Figure 3:**
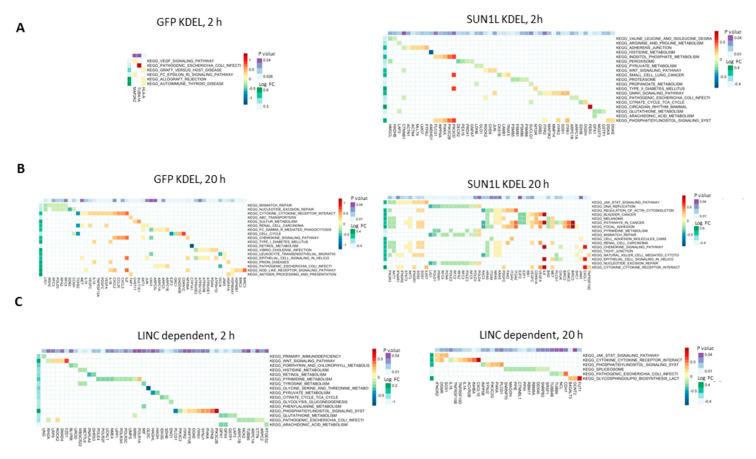
KEGG pathway analysis of differentially expressing genes in SMG vs. 1G after 2 and 20 h. (**A**) Pathways enriched with up-regulated and down-regulated genes in GFP-KDEL cells (left) and SUN1L-KDEL cells (right) after 2h exposure to SMG. (**B**) Up- and down-regulated enriched pathways in GFP-KDEL cells (left) and SUN1L-KDEL cells (right) after 20 h in SMG. (**C**) LINC-dependent up- and down-regulated enriched pathways in SMG after 2 (left) and 20 h (right).

KEGG (Kyoto Encyclopedia of Genes and Genomes) signaling pathway analysis of the differentially expressed genes revealed that in GFP-KDEL cells exposed to 2 h of SMG, six pathways were enriched, while a larger number of pathways were enriched only in the SUN1L-KDEL cells under the same conditions (Figure 3A). We next used the Molecular Signatures Database (MSigDB) to perform gene ontology analysis. The MSigDB is a curated set of annotated gene sets which can be analyzed by Gene Set Variation Analysis (GSVA) to determine if any genes in a particular curated set (pathway, biological state, cellular locale etc.) are enriched in a genome-wide expression analysis using a ranked set of genes [27,28]. Pathway analysis of the GFP-KDEL cells in SMG for the MSigDB GSVA GO biological process dataset revealed enrichment in genes related to microtubule-based movement, the reproductive process and negative regulation of catalytic activity (Supplementary Figure S5b). A similar analysis for cellular components revealed that terms relating to nuclear specks and the kinetochore were significantly enriched (Supplementary Figure S5a). A greater number of significantly enriched GO pathways were identified in the SMG SUN1L-KDEL expressing cells, including many biological processes related to mechanoresponse systems such as: cell matrix adhesion, regulation of cell migration and negative regulation of cell migration (Supplementary Figure S5b). GO pathway analysis of cellular components revealed enrichment related to the mitochondrion, actin cytoskeleton and cell adhesion. Some of the LINC-dependent GO cellular component pathways for mechanosensing identified by the interaction effect were mitochondrion, cell junction, intercellular junction, tight junction, kinetochore and nuclear specks (Supplementary Figure S5c).

### 2.3. LINC Dependency of SMG-Induced Changes in Gene Expression Is Also Determined by the Duration of Exposure to SMG

After 20 h in SMG, the GFP-KDEL cells had a larger number of genes that were significantly differentially expressed compared to the 2 h condition. Additionally, there was an even larger number of differentially expressed genes in SUN1L-KDEL expressing cells (1279 upregulated, 1542 downregulated, *p* ≤ 0.05) than the GFP-KDEL cells (813 upregulated, 846 downregulated, ≤0.05) after 20 h (Figure 2C,D, Supplementary Tables S4 and S5). In GFP-KDEL cells, the levels of expression of several genes associated with cytokines and chemokines were significantly altered, including the VEGFA gene. While the focal adhesion associated genes TLN2 and SERPINE1 (involved in the regulation of cell adhesion and spreading) were upregulated, the ECM genes TIMP3 and LAMA5 were down-regulated. The expression of the NE gene encoding for nesprin-3 (SYNE3) showed a significant decrease in expression (Figure 2C). We identified the genes whose expression depended on the duration of exposure to SMG by assessing the interaction effect between GFP-KDEL transcriptional changes at 2 and 20 h (Supplementary Table S7).

We also identified the unique genes and pathways that depend on the LINC complex to potentially mechanosense the gravitational forces in SMG after 20 h by evaluating the interaction effect between SUN1L-KDEL and GFP-KDEL transcriptional changes at 20 h (Figure 2F, Supplementary Figure S3F, Supplementary Table S6). All the above-mentioned genes were differentially expressed in SMG-treated SUN1-KDEL cells, in a similar fashion to GFP-KDEL, implying that they were not dependent on the LINC complex, with the notable exception of TIMP3. In contrast, MMP19 (encodes a collagenase related to ECM dynamics), which was not responsive to SMG in GFP-KDEL cells, gained responsiveness upon LINC complex disruption. Similarly, the chemokine gene CXCL16 was also modulated differently in SMG-treated SUN1-KDEL cells compared to the expression in GFP KDEL cells (Figure 2C,D). We also identified the genes in SUN1L-KDEL cells that were dependent on the duration of exposure to SMG by assessing the interaction effect between SUN1L-KDEL transcriptional changes at 2 and 20 h (Supplementary Table S8).

More KEGG pathways were enriched in GFP-KDEL cells after 20 h in SMG when compared to the effects of 2 h exposure (Figure 3A,B). For example, the cell cycle pathway was enriched in the differentially expressed transcripts in GFP-KDEL cells exposed to SMG for 20 h, whereas it was not affected in cells exposed for 2 h. In the SUN1-KDEL cells, regulation of the actin cytoskeleton was highlighted in the KEGG analysis, as were pathways associated with adhesion, such as focal adhesion, cell adhesion molecules and tight junction, implying alteration in the systems mediating cell-to-matrix and cell-to-cell contacts in SMG. The DNA replication mismatch repair pathway was also enriched, which may be linked to alterations in various cancer associated pathways/processes highlighted as being differentially regulated in the data (bladder cancer, melanoma, pathways in cancer, renal cell carcinoma). Altered cytokine receptor interaction and chemokine signaling pathways were also seen in this analysis. Thus, the LINC complex disruption in simulated microgravity may be associated with pathways related to cancer development. We also identified the KEGG pathways that are LINC-dependent in microgravity with the interaction model. It is important to note that there were a greater number of LINC-dependent pathways that were enriched at 2 h when compared to 20 h (Figure 3C). 

**Figure 4:**
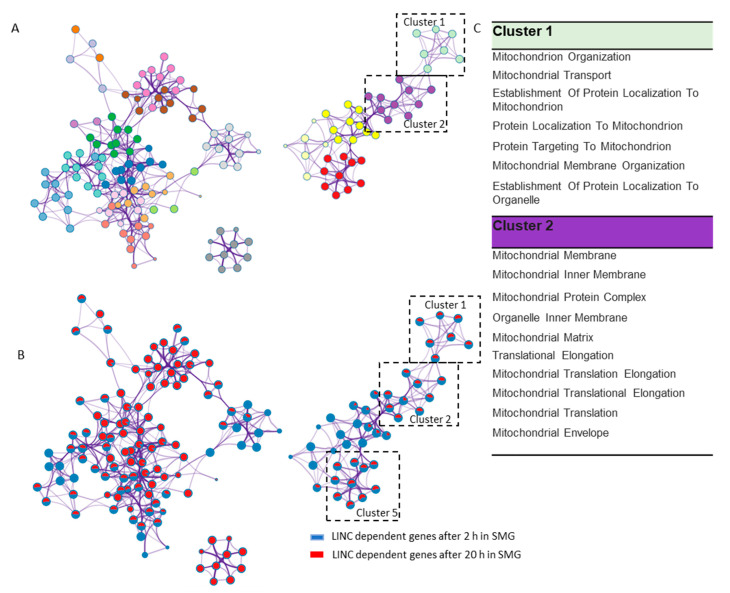
Network of top 20 clusters of the enriched terms. (**A**) Network representing each cluster by a different color; (**B**) network enrichment shown with pie chart. The size of the pie in each node depends on the gene count of the two conditions. Red indicates enrichment of pathways due to LINC-dependent genes at 20 h of SMG, and blue indicates enrichment of pathways in LINC-dependent genes at 2 h of SMG. (**C**) Enriched terms in Clusters 1 and 2 are all linked to mitochondria.

Cluster analysis was next performed on the LINC-dependent differentially expressing genes (*p* ≤ 0.05) at both 2 and 20 h SMG exposure. Using the Metascape tool (www.metascape.org), pathway and process enrichment analysis was performed using KEGG Functional Sets, KEGG Pathway, in this approach, network and clusters were developed based on the enrichment with *p* ≤ 0.01 and with a minimum count of 3. In Figure 4A, the top 20 clusters are shown with each node color coded based on the cluster it belongs to. Each node represents an enriched term as detailed in Supplementary Table S9. From the pie chart representation of each node (Figure 4B), we can see that clusters 1, 2 and 5 had all the nodes enriched for both the LINC-dependent 2 and 20 h SMG exposure conditions. Interestingly, Cluster 1 and Cluster 2 comprised enriched terms all related to the mitochondrian as listed in Figure 4C. Previous studies have found similar dysregulation and dysfunction of the mitochondrian in human cells exposed to simulated microgravity provided by 3-D clinostat and RPM exposures [29,30]. Our analysis clearly shows that the LINC complex may be required in regulating mitochondrion-dependent pathways and processes due to simulated microgravity at 2 and 20 h. We hypothesize that LINC may be playing a crucial role in modulating the mitochondrial response to SMG. However, more experimentation will be required to robustly test how LINC may be impacting the response of mitochondria in simulated microgravity. 

The GO pathway analysis of these data revealed that processes such as regulation of cell growth, cell activation and protein folding were enriched, alongside cellular components including the actin cytoskeleton, myosin complex, microtubule organization center and endoplasmic reticulum in GFP-KDEL cells (Supplementary Figure S6a,c). The GO analysis for of SUN1L-KDEL cells further revealed enrichment in biological processes such as response to external stimulus, response to extracellular stimulus, response to hypoxia, response to wounding and inflammatory response (Supplementary Figure S6d). The GO analysis for cellular components revealed a mix of upregulated and down-regulated processes after 20 h of SMG (Supplementary Figure S6b): for example, upregulation of actin cytoskeleton, leading edge, cell junction, tight junction and downregulation of microtubule organizing center and chromosomes. Some patterns of gene expression were consistent between responses at 2 and 20 h, including enrichment in cell adhesion and the actin cytoskeleton. The GO cellular component pathway analysis with the interaction model revealed the LINC-dependent components after 20 h in SMG mainly highlighted the tight junction, plasma membrane, intercellular junction components and mitochondrial components. The LINC-dependent GO biological pathways were also identified (Supplementary Figure S6c).

## 3. Discussion

A highly reproducible suite of effects were observed in cells exposed to altered gravity relating to the reorganization of their cytoskeleton and cell adhesion proteins [31], coupled with changes in the morphologies of the cells, including their nuclei. There is a close link between nuclear shape and the cytoskeleton, with various cytoskeletal elements playing roles in regulating nuclear shape and position. Thus, actin polymerization flattens the nucleus and repositions it by pulling on it. Additionally, forces generated by microtubule motor proteins such as dynein and kinesin, and acto-myosin contractile and tensile forces are also transmitted to the nuclear surface. Intermediate filaments also form a cage-like structure around the nucleus giving it structural stability. Each of these elements are mechanically connected to the nucleus through LINC complex proteins. The mechanical integration afforded by these connections through the LINC complex plays a critical role in transferring mechanical signals from the ECM and the forces generated within the cytoplasm to the nucleus [32]. These signals regulate features such as nuclear shape, homeostatic position and rotation—all important elements for nuclear functioning. Therefore, not surprisingly, the LINC complex is important for an array of normal cellular functions such as migration, division, proliferation, differentiation and wound healing [33]. Consistent with these ideas of a central role of the LINC complex in mechanical signaling, there are also major LINC-associated diseases such as nuclear envelopathies, laminopathy, skeletal muscular dystrophy and cardiomyopathy. Abnormal regulation of LINC complex genes SUN1, SUN2 and nesprin 2 is also seen in breast cancer.

Several LINC-associated proteins such as SUN1, SUN2, Emerins and Nesprin-3 were seen to be differentially regulated in cancer cells exposed to simulated microgravity [13]. Indeed, SMG promotes breast cancer cell proliferation, invasion and adhesion, nuclear positioning, as well as cell cycle progression, apoptosis [22] and abnormal expression of cancer inducing cytokines [14]. The LINC complex is known to play a very important role in the normal formation and development of breast cell acinus and in regulating the shape of the cell and the nucleus in both the breast cell culture of single cells and of monolayers [24]. Thus, understanding how SMG affects these cellular processes holds potential for both understanding how gravitational forces might be playing roles in normal tissue development on Earth and in revealing potential practical issues and risks related to long duration spaceflight.

In the present study, we looked at breast epithelial cells (MCF 10A) in SMG with and without LINC disruption. We discovered that the changes in nuclear shape seen after 2 h and 20 h exposures to SMG depend on the LINC complex. Previous studies have shown that both the shape of cells and their nuclei are impacted by altered gravity environments [34], and our studies now provide evidence that this effect depends on a functional LINC complex. Thus, after 2 h in SMG, LINC disruption leads to a lack of nuclear shape change normally observed in cells with intact LINC function. After 20 h in SMG, the response reverses in direction, with the nucleus rounding up compared to flatter nuclei in cells with an intact LINC complex. As discussed earlier, the cytoskeleton reorganizes in microgravity, with disruption of the microtubule network, and a so-called ‘loosening’ of the cytokeratin network around the nucleus, and this may be the reason for the rounding up of the nucleus (increased nuclear height and decrease in x-y area) in GFP-KDEL control cells exposed to 2 h of SMG. We hypothesize that, as in SUN1L-KDEL cells, the cytoskeleton is not mechanically connected to the nuclear surface, and so such SMG-induced changes in the cytoskeleton are not able to affect the nuclear shape. After 20 h in SMG, however, the nuclear height decreased, and the x-y nuclear area increased in GFP-KDEL cells compared to the cells in 1G. This observation can be explained by the increase in cell area reported in both simulated microgravity and true microgravity experiments conducted for long durations, and that the cell shape is inextricably linked to the nuclear shape, i.e., cell spreading is driving nuclear “flattening” [24,35,36,37]. On the other hand, the SUN1L-KDEL cells showed an increase in nuclear height under the same conditions, consistent with the idea of a loss of cytoskeletal interactions that normally pull on the periphery of the nuclear envelope. It has been reported previously that the response of cell morphology to calibrated physical external stimulus in LINC disrupted cells is different when compared to the cells with intact LINC complex function [1,2]. Therefore, we may expect the nuclear morphology in the LINC-disrupted cells to also respond to the longer duration SMG very differently to the control GFP-KDEL cells. Despite these time-dependent effects, our analysis shows that both at 2 and 20 h SMG exposure times, the LINC complex plays a role in the nuclear shaping mechanism. These results support the idea that the mechanosensitivity of the nuclear shape to external stimuli strongly depends on the SUN1-Nesprin linkage in the nuclear envelope provided by the LINC complex [3]. 

We also looked at the role of the LINC complex in differential gene expression in response to SMG, revealing that there are LINC-dependent genes that are up- and down-regulated in response to SMG. We hypothesize that the nuclear shape regulation and gene expression dependency on the LINC complex are inter-related. Thus, gene activity depends on the location of the chromosome and its packing inside the nucleus [38], e.g., chromosome territories are positioned differently and have different interactions in breast epithelial cells (MCF 10A) compared to corresponding malignant cells (MCF 10CA1a), supporting different patterns of gene expression [39]. Nuclear shape and rigidity depends on the packing of the heterochromatin [40] and changes in nuclear shape may in turn feedback to change the level of chromatin condensation [41]. Therefore, it is highly likely that patterns of gene expression depend to some degree on nuclear shape [4,42]. The LINC complex connects the cytoskeleton to the nuclear surface; the SUN proteins in the inner nuclear envelope are connected to lamins, which are connected to chromatin. Thus, the LINC complex provides a strong candidate for linking cellular level mechanical forces to changes in nuclear gene expression. For example, interfering with the LINC complex in plant cells led to decondensation of chromocenters and silencing of transcription [43]. Hence, the LINC complex may impact gene expression in two ways: (1) by directly affecting chromatin compaction; (2) by regulating the shape of the nucleus, and both mechanisms may be in play once cells move from the 1G to a microgravity environment.

It is important to note that there are changes in cell physiology just with time growing in culture. For example, Seaman et al. have found that the nuclear morphology changes significantly over time in cultured human primary fibroblast cells [44]. Indeed, in our data both the GFP-KDEL and SUN1L-KDEL cells at 1G (Table 1) show expected decrease in nuclear area and increase in nuclear height when comparing data from 2 h and 20 h in culture. In most cell types, the shape of the nucleus depends mainly on factors such as the shape of the cell, cytoskeleton organization, and external adhesions such as cell-matrix and cell-cell adhesion [3,24,45]. Studies with human breast epithelial cells found that the shape of the nucleus is controlled by the shape of the cell [24]. Also, fibroblast cell nuclei are flatter in cells with larger spreading area than in lesser spread cells [37]. The dynamic actin cap flattens the nucleus while actomyosin contractile forces pull on the nucleus regulating its volume and the gross morphology [2,46,47]. Intermediate filaments also wrap the nucleus like a cage and act as a passive network that transmits active forces from myosin motor proteins thereby playing a significant role in controlling the nuclear volume [3,46,47]. While active motor forces and changes in lamellipodia and filopodial formation occur during cell migration [48,49], cell polarization [50], cell division [51,52] and development [53]. Each of these processes are dynamic and require regular nuclear positioning and shaping which in turn depend on the cell shape and cytoskeletal organization over time to carry on with normal cell functions. These observations suggest physiological changes occurring in the cells with time in culture indeed have the potential to affect the nuclear shape in addition to any nuclear morphological changes mediated by SMG. Both the GFP-KDEL and the SUN1L-KDEL expressing cells showed similar culture time-related effects, with e.g., nuclear shape changing in an identical fashion when comparing the 2 h and 20 h treatments at 1G. Therefore, to better control the physiological changes along the culture time, we particularly evaluated the differences between nuclear shape and patterns of gene expression in SUN1L-KDEL expressing cells relative to the paired GFP-KDEL control at the same duration in culture under 1G or SMG. Comparing the SMG responses in the SUN1L-KDEL cells to the paired SMG-treated GFP-KDEL controls at the same time-point helps remove these culture time-related effects to focus on the effects of SMG exposure.

Cancer is a leading cause of death around the world and with an ever-increasing range and duration of deep space exploration missions, astronauts are at a potentially elevated risk of developing this disease. For decades, cells have been studied in altered gravity environments using various platforms such as the International Space Station, sounding rockets, parabolic flights and microgravity simulators. Common cell functions such as proliferation, migration, division and differentiation are all affected differently in altered gravity environments [13,36,54,55]. Genes associated with the progression of cancer by metastasis (cytokines) are also differentially regulated in microgravity as are several other genes related to wound healing, focal adhesion [13], and growth factors. However, our analysis suggests a complex pattern of gene expression responses in microgravity in relation to the LINC complex. We discovered genes that are LINC-dependent in SMG that are associated with different types of cancer. For example, we found the gene RBMS3 to be significantly upregulated in a LINC-dependent manner in response to 2 h of SMG. This gene acts in a defense mechanism against breast and other types of cancer, and upregulation of this gene can suppress cancer or tumor progression [56]. Overexpression of the SRA1 gene is also associated with breast, ovarian, prostate, lung and liver cancer [57]. In our study, we found that SRA1 gene expression is sensitive to SMG. However, although the LINC-dependent SMG responsiveness of, e.g., RBMS3 remained at 20 h of SMG, it was lost for the SRA1 gene. These observations suggest an interplay of signaling and time in the LINC mediated modulation of these genes in response to SMG, and to potential effects on cancer development.

In conclusion, we discovered that the LINC complex plays an important role in regulating nuclear morphology in breast epithelial cells exposed to SMG. We identified a series of LINC-dependent SMG responsive genes, where the spectrum of changes depend on how long the cells are exposed to SMG. Using KEGG and GO analysis, we found that pathways and processes associated with the cell cycle, the cytoskeleton and cell adhesion were the most affected upon LINC disruption in SMG. Taken together, our results imply that the mechanical forces on a cell imposed through altered gravity are likely acting through LINC complex-related transduction events to modulate both nuclear shape and patterns of gene expression, especially of genes associated with the cytoskeleton, cell adhesion and other mechanical features of the cell. These results provide insight into a potentially important element of transcriptional control on Earth and highlight a potential risk factor for long duration spaceflight. Further studies using both true and simulated microgravity need to be performed to fully elucidate the molecular mechanisms behind LINC in cellular sensing and the response to microgravity.

## 4. Materials and Methods

### 4.1. Cell Culture

Human breast epithelial cells (MCF 10A from ATCC), MCF 10A SS-GFP-KDEL and MCF 10A SS-GFP-SUN1L-KDEL cells (created by Dr. Tanmay Lele and Dr. Kyle Roux [21,25]) were a kind gift to us. The inducible cell lines were created with MCF 10A TET-ON (Clontech) transduced retrovirally with pRetroX-Tight.puro containing either SS-HA-GFP-SUN1L-KDEL for LINC disruption or SS-HA-GFP-KDEL for controls [25]. To create a stable line, cell selection was performed with 0.5 μg/mL puromycin and then screened by immunofluorescence postinduction with or without 1 μg/mL doxycycline for 18 h. The cells were cultured in DMEM/F12 (Thermofisher, Grand Island, NY, USA) without phenol red, supplemented with 5% (*w/v*) horse serum, cholera toxin (Sigma, Saint Louis, MO USA), epidermal growth factor (Peprotech, Cranbury, NJ, USA), hydrocortisone (Sigma), insulin (Sigma) and penicillin streptomycin (Thermofisher, Grand Island, NY, USA). The cells were maintained at 37 °C in a humidified, 5% (*v/v*) CO_2_ incubating chamber. The inducible cells were trypsinized and seeded in fibronectin coated chamber flasks with 30% confluency and cultured for 24 h. To induce SUN1L-KDEL for LINC disruption and GFP-KDEL for control comparison, the media were supplemented with 1 µg/mL doxycycline [21]. These cells were cultured in doxycycline for 24 h before placing them on the 3-D clinostat. 

### 4.2. Simulated Microgravity Using 3-D Clinostat

The chamber flasks were filled completely with media containing 1 µg/mL doxycycline (Fisher) and care taken to remove all the bubbles that may cause shear stress to the cell monolayer. The chamber flask was sealed with a rubber stopper, and a syringe was used to release any excess pressure. The 3-D clinostat (Gravite, Space Bio Laboratories Ltd., Hiroshima, Japan) consisting of two perpendicular rotating axes was run in Mode C preprogrammed with an inbuilt computer and gravity acceleration sensor to measure simulate microgravity (to the order of 10^−3^ G) by rotating the inner and outer frame simultaneously at a constant angular velocity (~2 rotations per minute) [58,59,60]. Four cell culture flasks on slides (Thermofisher, Grand Island, NY, USA, 170920) were mounted at the center and were placed in a controlled environment maintaining the temperature at 37 °C for 2 and 20 h. The 1G control samples were placed in the same incubator. All the experiments were conducted twice, and each experiment had two sets of samples in both 1G and SMG conditions. A Student’s *t*-test was applied for statistical comparison for nuclear morphology.

### 4.3. Immunostaining and Microscopy

After the cells were exposed to simulated microgravity for fixed periods of time, they were fixed with 4% paraformaldehyde for 10 min and then washed with PBS. To stain for F-actin, 1:50 Alex Fluor 594 phalloidin was used and for the nucleus, 1:250 Hoechst 33,342 (Life Technologies, Carlsbad, CA, USA) was used. Cells were then incubated for 20 min at room temperature before washing with PBS. The cells were then imaged with the 60×/1.40 NA oil immersion objective of a Nikon A1R laser scanning inverted confocal microscope (Nikon Instruments, Melville, NY, USA). To immunostain for the Nesprin-2 protein, we followed the protocol of Zhang et al. [21] and used the primary antibody anti-Nesprin-2 (Abcam, Cambridge, MA, USA, ab204308) at a 1:100 dilution and the secondary antibody goat anti-rabbit IgG (Thermofisher, Grand Island, NY, USA, A-11037) at a 1:200 dilution. Z-stack images were acquired for the cells with 0.3 µM height intervals. The images were reconstructed using the Nikon NIS elements software, and maximum intensity profiles for the nucleus were created for the x-z view. The full width half maximum (FWHM) technique in MATLAB (MathWorks, Natick, MA, USA) was used to acquire the nuclear height measurement. The maximum intensity projection of the x-y view of the nucleus was used to measure the nuclear area using the ImageJ software.

### 4.4. RNA seq Library Preparation, Sequencing and Data Analysis

Approximately 2 × 10^6^ cells were frozen in 700 µL of QIAzol buffer (Qiagen, Hilden, Germany). The samples were immediately vortexed and placed on ice before storing at −80 °C. Once all samples were collected, they were randomized, and total RNA was extracted and purified using the Qiagen RNeasy Micro Kit, following recommended procedures, with on-column DNase treatment. Libraries were prepared from purified RNA (0.3 ng) using the TruSeq Stranded Total RNA Library Prep Globin (Illumina, San Diego, CA, USA) according to the manufacturer’s protocol. Three runs of 75 bp paired-end sequencing were performed on pooled libraries using the Illumina NextSeq 550 System. Quality control of purified RNA and RNA libraries was carried out using the Fragment Analyzer System (Agilent Technologies, Santa Clara, CA, USA).

Raw DE multiplexed fastq paired end read files were trimmed of adapters and filtered using the program skewer [61] to discard any reads with an average phred quality score of less than 30, or a length of less than 36 nucleotides. Trimmed reads were then aligned using the HISAT2 aligner to the *Homo sapiens* NCBI reference genome assembly version GRCh38 and sorted using SAM tools [62,63]. Aligned reads were counted and assigned to gene metafeatures using the program feature Counts as part of the Subread package [64]. These count files were imported into the R programming language and were assessed for quality control, normalized and analyzed using an in-house pipeline utilizing the edge R Bioconductor library, for differential gene expression testing, and the GSVA library, for GO and KEGG pathway analysis, utilizing their respective collections from the Molecular Signatures Database (MSigDB) [28,65,66]. The GO pathway analysis shown in Supplementary Figure S5 is based on GSVA using the Molecular Signatures Database (MSigDB) GO collections, as opposed to the GO term enrichment, which does not take into account the ranking of the genes [27,28].

## Figures and Tables

**Table 1:** Nuclear morphology: After 20 h exposure to SMG, the nuclear height decreased in GFP-KDEL cells, while the height increased in SUN1L-KDEL cells again indicating a role of the LINC complex in the response of the nuclear shape to SMG. * *p* < 0.05, comparing same cell line to the 1G condition within the same time-point, statistically significantly different; the comparisons are with the corresponding 1G control. All values are shown as the mean ± SD.2.2. Short Duration Simulated Microgravity-Induced, LINC-Dependent Changes in Nuclear Shape.

	Cell Type	GFP-KDEL			SUN1L-KDEL		
time		n	Nuclear height (µm)	X-Y nuclear area (µm^2^)	n	Nuclear height (µm)	X-Y nuclear area (µm^2^)
20 h	Ground	74	2.7 ± 1.1	207 ± 89	73	2.7 ± 1.2	216 ± 111
	SMG	81	2.2 ± 1.1 *	267 ± 148 *	99	3.4 ± 1.1 *	164 ± 41 *
2 h	Ground	82	2.0 ± 0.8	234 ± 104	45	2.2 ± 0.6	212 ± 60
	SMG	68	3.0 ± 1.5 *	176 ± 107 *	43	2.6 ± 1.1	193 ± 60

## Supplementary Materials

The following are available online at https://www.mdpi.com/1422-0067/21/18/6762/s1.
