# Supplementary material for: Changes in Nuclear Shape and Gene Expression in Response to Simulated Microgravity Are LINC Complex-Dependent
Source: Int J Mol Sci. 2020 Sep 15;21(18):6762. doi: 10.3390/ijms21186762 (PMC7555797; doi:10.3390/ijms21186762)
Supplement: Supplementary file 1 [file ijms-21-06762-s001.zip › New files/Figure_All_0902202_supplementary.pdf]

Supplementary Figure 1

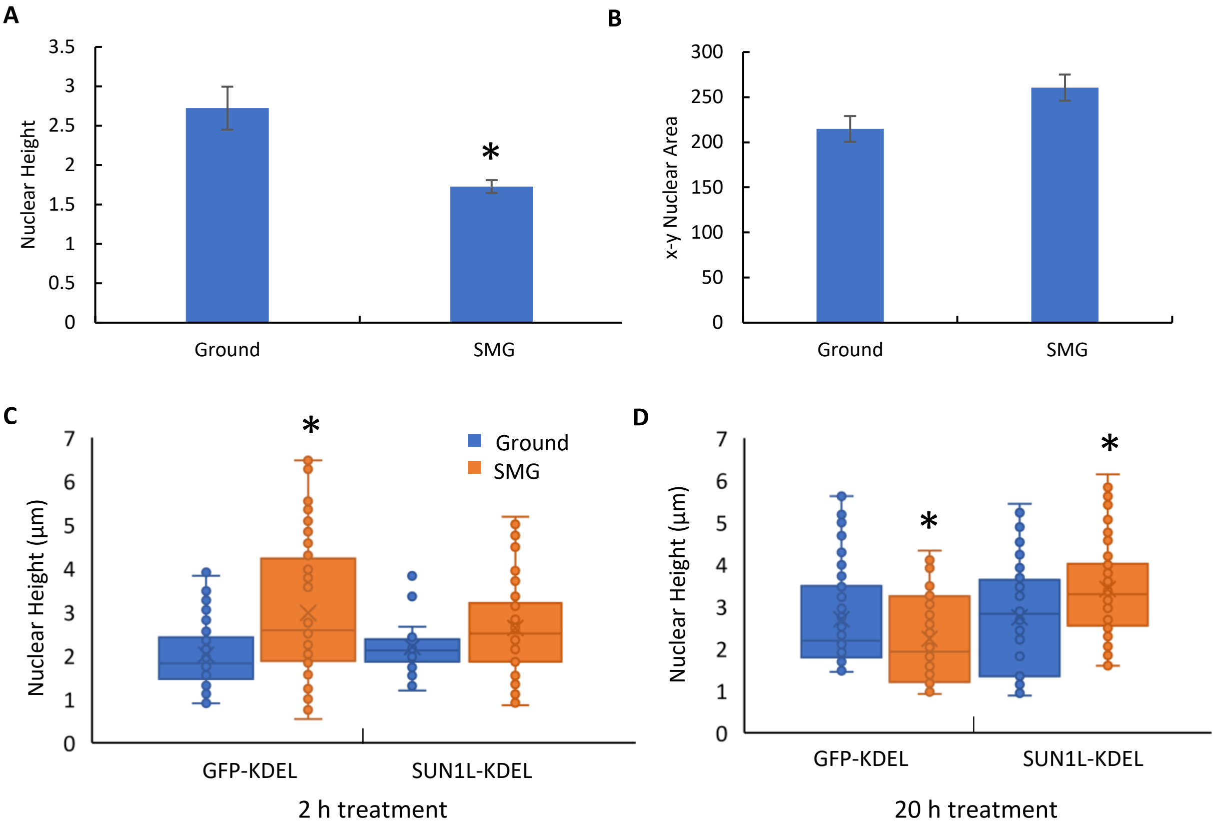

Nuclear shape response to simulated microgravity (SMG) in wild-type MCF 10A cells. (A) Nuclear height was significantly decreased after 20 h exposure to SMG ($p \leq 0.05$). (B) the nuclear spreading area in x-y plane was also consistent with the nuclear height, while no significant difference was. n=107. (C)) Nuclear height was significantly larger in GFP cells exposed to SMG for 2 hr reflecting the response of the nuclear shape to SMG, while no difference was observed in SUN1L cells. (D) After 20 hr exposure to SMG, the nuclear height decreased in GFP cells while the height increased in SUN1L cells again indicating the role of LINC complex in the response of the nuclear shape to SMG. *$p<0.05$; the comparisons are with the corresponding 1G control. All values are shown as a mean ± SEM

Supplementary Figure 2

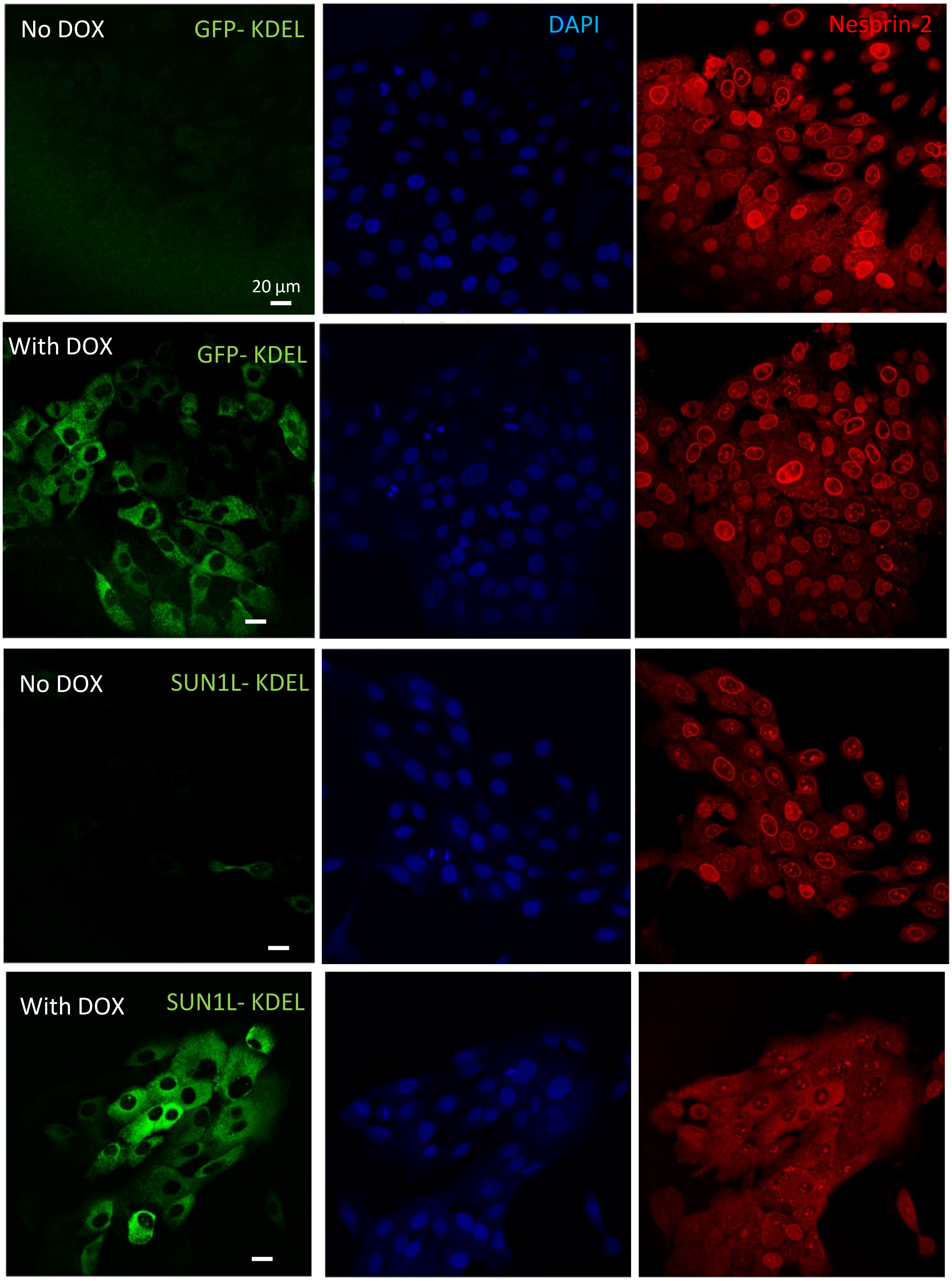

Doxycycline inducible LINC disruption: Immunofluorescence images MCF 10 SS-HA-GFP-KDEL (GFP-KDEL) and SS-GFP-SUN1L-KDEL (SUN1L-KDEL) show the expression of GFP upon inducing with Doxycycline (DOX) and the nuclei stained with Hoechst dye. The SUN1L-KDEL cells when induced with Doxycycline (With DOX) show almost no expression of Nesprin-2 (red) in the nuclear envelope (NE) while cells with no DOX show a clear ring of Nesprin-2. GFP-KDEL cells show Nesprin-2 in the NE with and without DOX.

Supplementary Figure 3

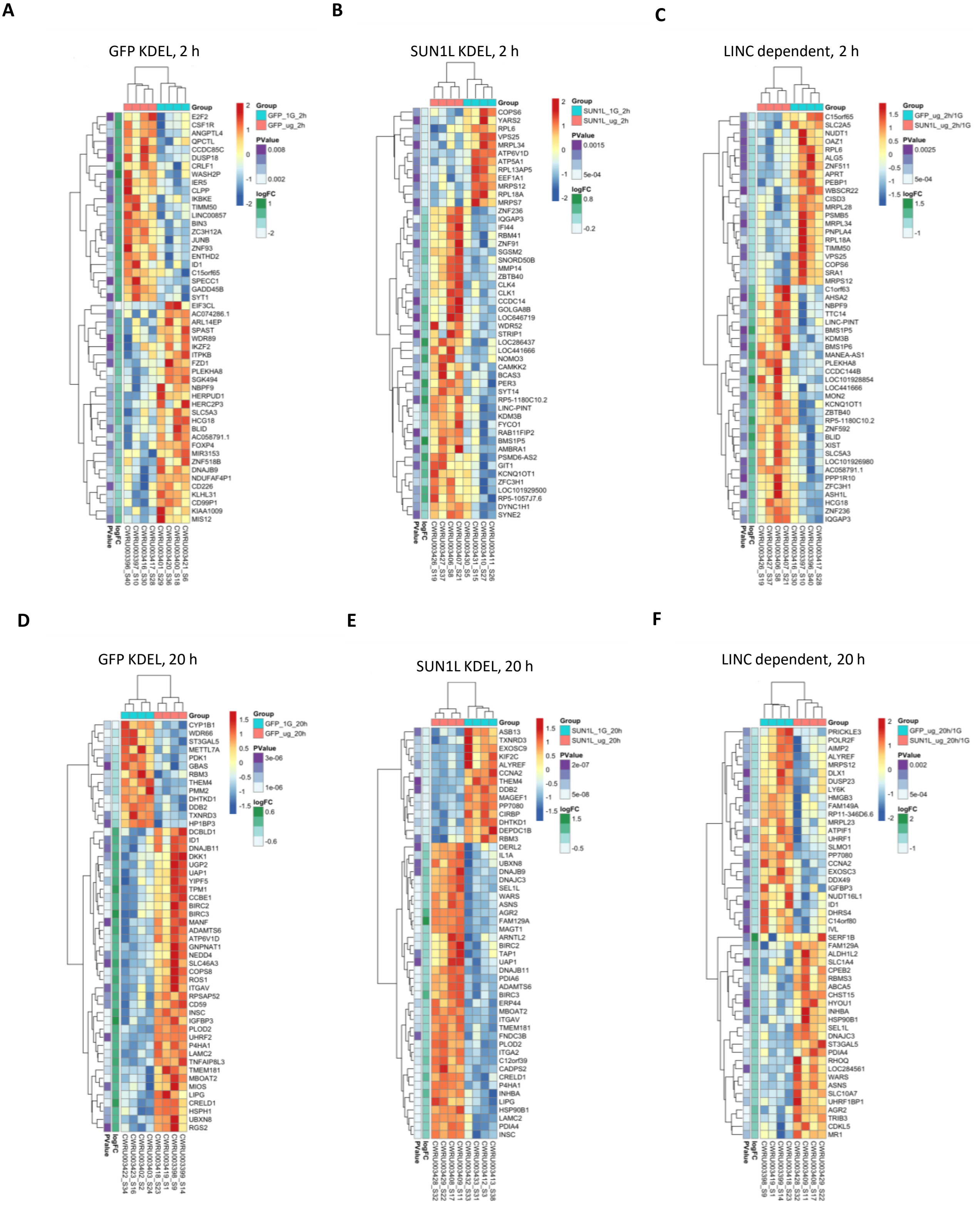

Top 50 differentially expressing genes

Supplementary Figure 4

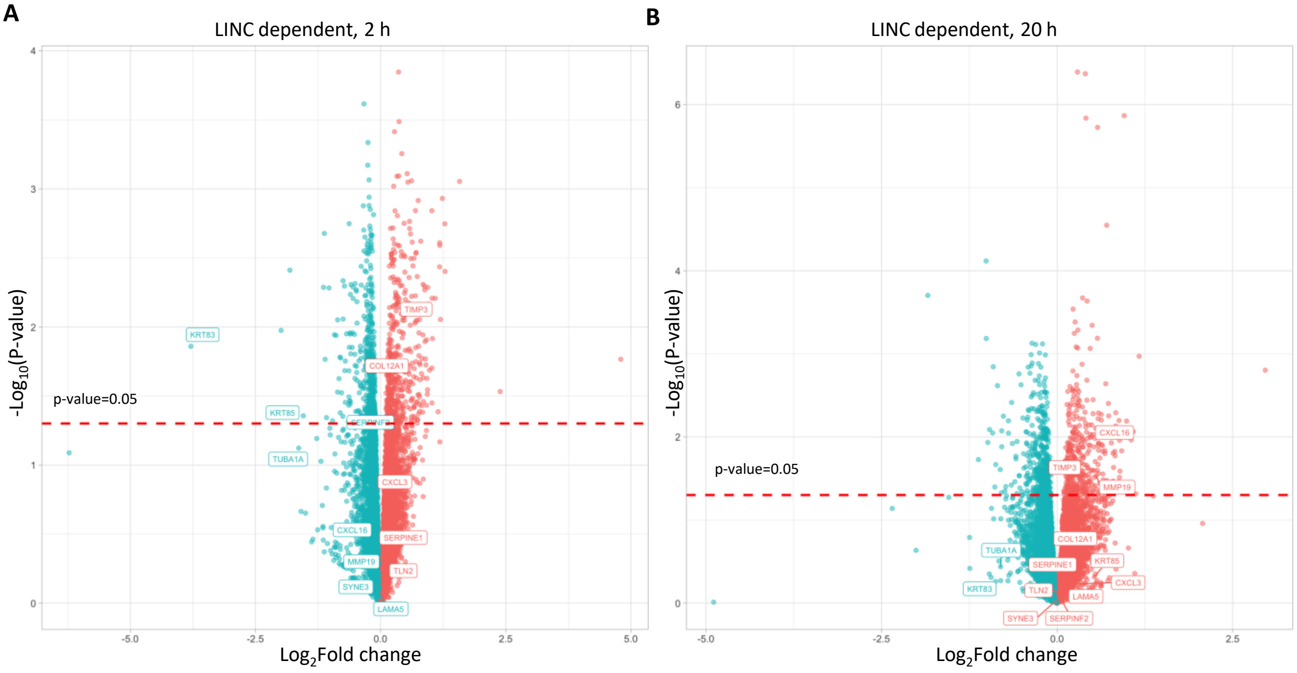

LINC dependent differential gene expression. Volcano plots of differentially expressing genes between SMG and 1G with statistical significance in the y-axis and fold change on the x-axis after 2 h and 20 h in SMG. Upregulated genes are shown in orange and downregulated genes are shown in blue. Significantly differentially expressing genes with p≤0.05 are above the red dashed line.

Supplementary Figure 5

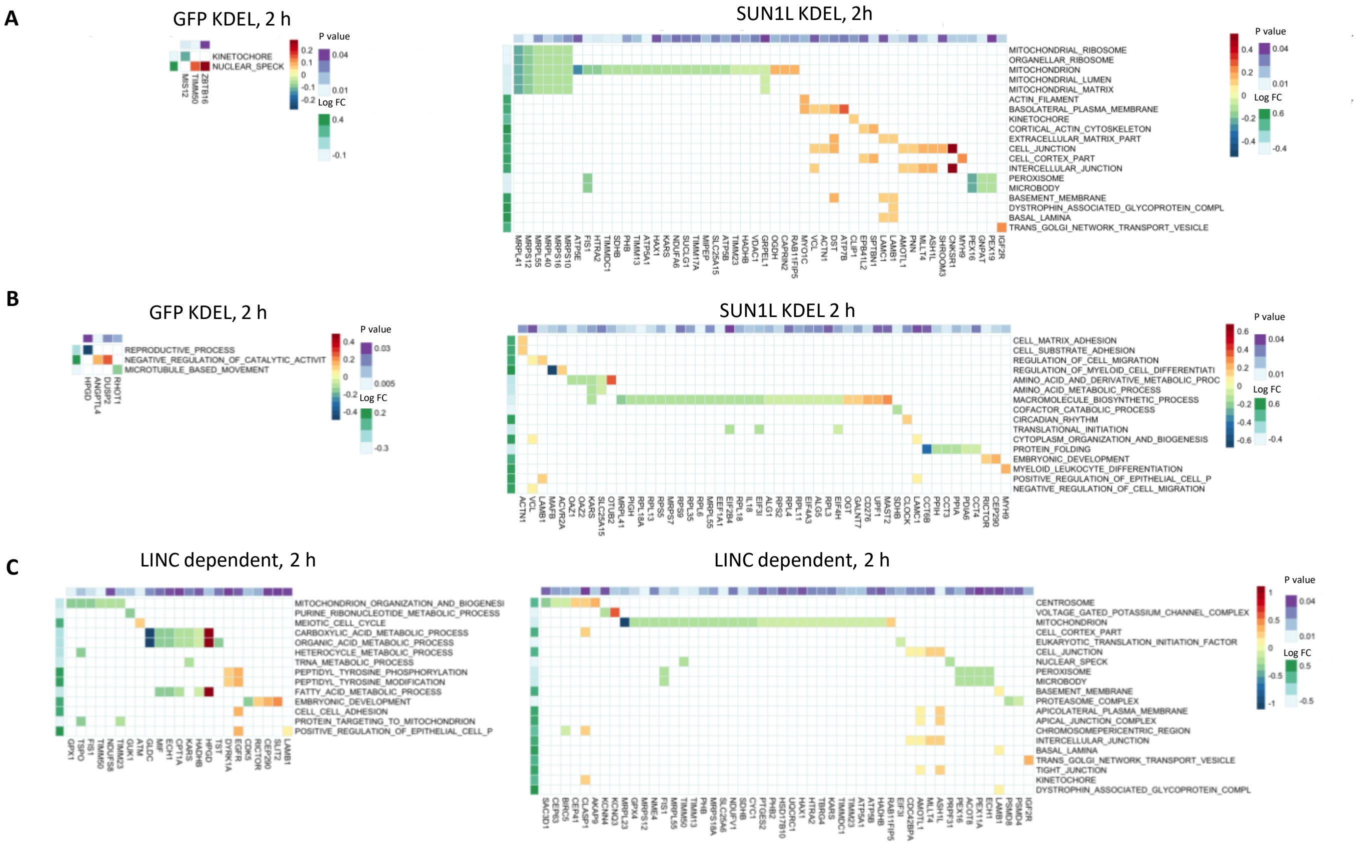

Gene Ontology (GO) analysis of differentially expressing genes in two ontologies in SMG vs 1G after 2 hours. (a) up regulated and down regulated genes enriched for cellular component in GFP KDEL cells (left) and SUN1L-KDEL cells (right). (b) Up and down regulated genes enriched for biological process in GFP KDEL cells (left) and SUN1L-KDEL cells (right). (c) LINC dependent up and down regulated genes and pathways enriched for biological process (left) and cellular component (right) in response to SMG identified by interaction effect.

Supplementary Figure 6

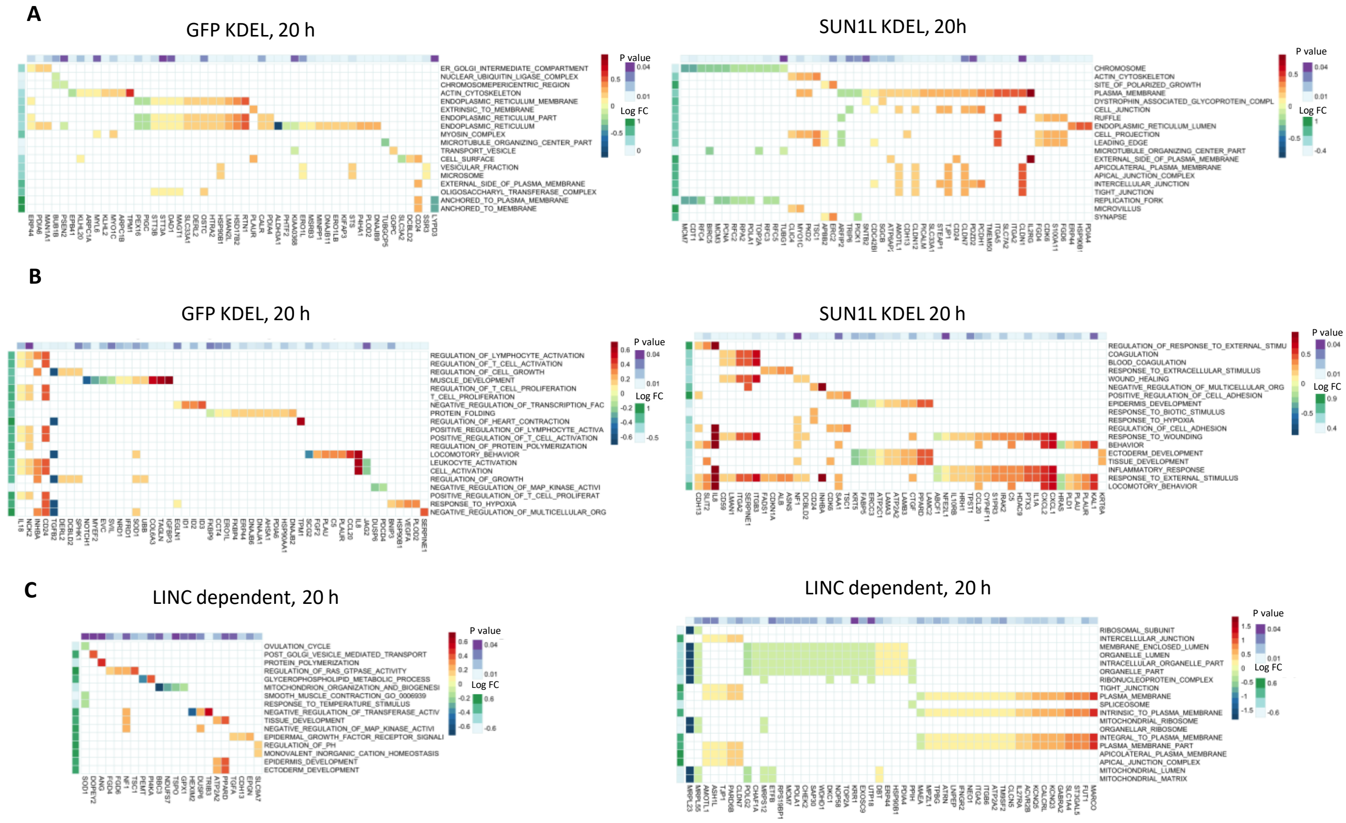

Gene Ontology (GO) analysis of differentially expressing genes in two ontologies in SMG vs 1G after 20 hours. (a) up regulated and down regulated genes enriched for cellular component in GFP KDEL cells (left) and SUN1L-KDEL cells (right). (b) Up and down regulated genes enriched for biological process in GFP KDEL cells (left) and SUN1L-KDEL cells (right). (c) LINC dependent up and down regulated genes in SMG enriched for biological process (left) and cellular component (right).
